# Vascular Damage in Resistant Hypertension: TNF-Alpha Inhibition Effects on Endothelial Cells

**DOI:** 10.1155/2015/631594

**Published:** 2015-10-04

**Authors:** Natália Ruggeri Barbaro, Thiago Matos de Araújo, José Eduardo Tanus-Santos, Gabriel Forato Anhê, Vanessa Fontana, Heitor Moreno

**Affiliations:** ^1^Laboratory of Cardiovascular Pharmacology, Faculty of Medical Sciences, University of Campinas, 13084971 Campinas, SP, Brazil; ^2^Department of Pharmacology, Faculty of Medical Sciences, University of Campinas, 13084971 Campinas, SP, Brazil; ^3^Department of Pharmacology, Ribeirao Preto Medical School, University of Sao Paulo, 14049900 Ribeirao Preto, SP, Brazil

## Abstract

Inflammatory cytokines have been associated with the pathophysiology of hypertension and target organ damage (TOD). Resistant hypertensive patients (RHTN) are characterized by poor blood pressure control and higher prevalence of TOD. This study evaluated the relationship between plasma levels of TNF-*α* and arterial stiffness (pulse wave velocity—PWV) in 32 RHTN and 19 normotensive subjects. Moreover, we investigated the effect of TNF-*α* inhibition on human endothelial cells (HUVECs) incubated with serum from RHTN and normotensive subjects. HUVECs containing serum obtained from normotensive (*n* = 8) and hypertensive (*n* = 8) individuals were treated with TNF-*α* inhibitor (infliximab). Cell suspensions were used for measurement of DNA fragmentation and reactive oxygen species (ROS) content. RHTN patients showed higher levels of TNF-*α* compared to normotensive subjects, as well as higher PWV. Positive correlation was found between TNF-*α* levels and PWV measures in the whole group. HUVECs incubated with serum from RHTN showed increased cell apoptosis and higher ROS content compared to normotensive subjects. Infliximab attenuated the apoptosis of HUVECs incubated with serum from RHTN, but no effect in ROS production was observed. Our findings suggest that TNF-*α* might mediate, at least in part, vascular damage in resistant hypertension.

## 1. Introduction

Several studies have demonstrated the participation of inflammatory cytokines in the genesis of hypertension in humans [1, 2] and animal models [3, 4]. Mice lacking T cells (RAG-1^−/−^ mice) showed attenuated hypertension after angiotensin II infusion and desoxycorticosterone acetate- (DOCA-) salt or norepinephrine administration [3, 5]. Moreover, increased secretion of cytokines such as IFN-*γ*, IL-17A, and TNF-*α* by circulatory spleen-derived T cells was observed in Ang II-induced hypertension. In these animals, the inhibition of TNF-*α* prevented increased vascular superoxide production and hypertension mediated by angiotensin II [3]. Although it has been suggested that several cytokines are involved in vascular damage induced by hypertension, TNF-*α* inhibition decreases blood pressure and prevents target organ damage in animal studies [3, 6]. Also, the inhibition of TNF-*α* in humans showed BP reduction [7]. On the other hand, the role of this cytokine in hypertensive subjects has been poorly studied.

Resistant hypertensive patients (RHTN) represent extreme phenotype of hypertension, characterized by poor blood pressure control and higher prevalence of target organ damage, which explain the unfavorable prognosis associated with this condition [8]. Vascular injury is a frequent characteristic induced by hypertensive disease. Our group showed that RHTN have higher arterial stiffness and impaired endothelial function compared to normotensive and mild to moderate subjects [9]. Conductance vessels gradually show reduction in distensibility and compliance, phenomena known as arterial stiffness. Arterial stiffness is an independent predictor of all-cause and cardiovascular mortality in hypertensive patients and is characterized by structural changes in connective tissue proteins in the vascular wall [10]. In addition to extracellular matrix remodeling, oxidative stress and inflammatory markers are key players in vascular remodeling associated with hypertension. Angiotensin II stimulates NADPH oxidase activity, increases reactive oxygen species production (ROS), and reduces NO availability leading to endothelial dysfunction [11, 12]. In addition, ROS increases collagen secretion by vascular smooth cells [13], which may favor vascular stiffness. Both Ang II and ROS signaling activate cytokines production, including TNF-*α*, and the expression of cell adhesion molecules including VCAM-1 and MCP-1 [14, 15]. In turn, vascular inflammation stimulates vascular fibrosis and smooth muscle cells proliferation, subsequently increasing arterial stiffness [15]. Inflammatory markers such as TNF-*α*, C-reactive protein, and interleukin-6 are correlated with arterial stiffness in hypertensive subjects [16]. In fact, previous studies demonstrated that the infusion of angiotensin II in mice lacking T and B cells (RAG-1^−/−^) or interleukin-17a (IL-17a^−/−^) reduces collagen deposition in the aorta and superoxide production and preserved endothelium-dependent vasodilatation compared to wild type animals [17, 18]. Interestingly, human aortic smooth muscle cells treated concomitantly with IL-17 plus TNF-*α* showed increased expression of several genes related to vascular dysfunction and inflammation [18]. Taken together, these data demonstrated that T cells-derived cytokines may play a critical role in vascular stiffening.

We recently found that TNF-*α* inhibition with infliximab reduced systolic BP, left ventricular hypertrophy, and vascular inflammation in spontaneously hypertensive rats (SHR) [6]. Moreover, hypertensive subjects showed increased arterial stiffness and higher plasma levels of TNF-*α* compared with normotensive subjects [19]. TNF-*α* inhibition has been discussed as potential strategy to improve vascular function [20, 21]. On the other hand, the treatment with anti-TNF-*α* improves vascular endothelial function and decreases arterial stiffness in postmenopausal women [22] and rheumatoid arthritis patients [23].

Despite some studies suggesting TNF-*α* as a potential marker of vascular inflammation, the causal role of this cytokine in the pathogenesis of hypertension is underexplored. The association of TNF-*α* has been extensively reported in hypertension, but no previous study evaluated the effects of TNF-*α* on human endothelial cells. Thus, this study was designed to evaluate the relationship between plasma levels of TNF-*α* and arterial stiffness in RHTN and normotensive subjects. Moreover, we investigated the effect of TNF-*α* inhibition on human endothelial cells incubated with serum from RHTN and normotensive subjects.

## 2. Methods

### 2.1. Patient Population

This cross-sectional study was performed in the Outpatient Resistant Hypertension Clinic at the University of Campinas Hospital. Thirty-two patients classified as RHTN and 19 normotensive subjects were included in this study. Resistant hypertension (RHTN) was defined as blood pressure (BP) that remained above goals despite the concurrent use of 3 antihypertensive agents of different classes, including a diuretic, at optimal dose amounts. Also, patients whose blood pressure was controlled but required 4 or more medications were also considered resistant [24]. Hypertensive patients were followed up and treated for at least 6 months with regular scheduled appointments before being characterized as resistant to treatments. Exclusion criteria included secondary hypertension (identifiable and removable causes of hypertension, including Conn's or Cushing's syndrome, diabetes, renal artery stenosis, pheochromocytoma, and coarctation of the aorta), liver and renal disease, heart failure (ejection fraction < 50%), stroke, peripheral vascular disease, smokers, obesity (BMI ≥ 30 kg/m²), pregnancy or oral contraceptive use, history or clinical evidence of recent infection, and use of anti-inflammatory drugs.

### 2.2. Laboratory Assessments

Blood samples were collected at 8:00 a.m. after overnight fasting. The plasma levels of TNF-*α* were determined by ELISA (R&D Systems, Inc., Minneapolis, USA) according to manufacturer's instructions. Biochemical assessments, including serum cholesterol, LDL, HDL, triglycerides, glucose, aldosterone, and creatinine, were performed by central laboratory at the University of Campinas Hospital.

### 2.3. PWV Measurement

Pulse wave velocity was measured using the SphygmoCor System (Atcor Medical, Sydney, Australia) with the patient in the supine position. Pulse wave of the carotid and femoral arteries was determined by estimating the delay with respect to the electrocardiogram wave. A measuring tape was used to measure the distance from the sternal notch to the carotid-femoral recording site. Carotid-femoral PWV was calculated by dividing traveled distance by transit time (PWV = distance/time). At least two measurements were performed in each patient. The PWV value was reported as the mean and the values were corrected for mean arterial pressure.

### 2.4. Collection and Preparation of Serum Samples

At the study visit, blood was collected by antecubital vein puncture using serum-separating tubes (BD Vacutainer System). After 30 minutes of resting at room temperature, whole blood samples were centrifuged for 10 minutes at 4,000 rpm. Serum was stored at −80°C in 1 mL aliquots until the cytokine measurements.

### 2.5. Human Umbilical Vein Endothelial Cells (HUVECs) Cell Culture and Plasma Incubation Conditions

HUVEC cell lines (CRL-2873, ATCC, Manassas, VA, USA) were cultured at 37°C in 5% CO_2_ in Dulbecco's Modified Eagle's Medium (Vitrocell Embriolife, Brazil), supplemented with glucose 4,500 mg/L and 10% (v/v) fetal bovine serum (FBS). The cells were used at passage 3 for the experiments. HUVECs were plated in 6-well plates at a density of 2 × 10^5^ cells/well (for flow cytometry assays) and in 25 cm^2^ tissue culture flasks at a density of 4 × 10^5^ cells/flask (for gene expression experiments). After 24 hours, the medium was replaced by FBS-free medium containing 10% of serum obtained from normotensive (*n* = 8) and hypertensive (*n* = 8) individuals. The cells were incubated for additional 24 hours with the patients' serum. The experiments were performed in duplicate. When used, infliximab (400 *μ*g/mL) was added to the cells concomitantly with human serum.

### 2.6. Flow Cytometry Analysis of DNA Fragmentation and Reactive Oxygen Species (ROS) Content

The medium was discarded and adherent cells were washed with Krebs buffer. The cells were detached using a cell scraper. Cell suspensions were divided into two aliquots used for measurement of DNA fragmentation and ROS content.

For DNA fragmentation measurements, cells were centrifuged (2,000 ×g, 4°C for 10 min) and the pellets were resuspended in 250 *μ*L of DNA fragmentation buffer (PBS containing 0.1% Triton X-100, 8 *μ*g/mL propidium iodide, and 10 mg/mL sodium citrate) and incubated overnight at 4°C. After the incubation, samples were analyzed using a FACSCalibur flow cytometer (Becton Dickinson, San Juan, PR, USA). Fluorescence of 10,000 events was acquired in the FL2 channel and analyzed using the CellQuest software. The cells with fragmented DNA emitted lower fluorescence signal, compared to cells with intact diploid and tetraploid DNA, which emitted characteristic two-peaked high-intensity fluorescence. The percentage of apoptotic cells was calculated using the number of low fluorescence events.

For ROS content measurements, cells were incubated with 1 mL of Krebs buffer containing 5 mM 2,7-dichlorodihydrofluorescein diacetate (DCFH) (Sigma, catalog number: D6883) for 30 min at room temperature. After incubation, samples were analyzed using a FACSCalibur flow cytometer. Fluorescence of 10,000 events was acquired in the FL1 channel and analyzed using the CellQuest Pro software. Mean fluorescence was obtained from a M1 population defined in the histograms. The limits for the M1 population were set based on the fluorescence of the unstained cells.

### 2.7. Real-Time Reverse Transcription Polymerase Chain Reaction

The total RNA was extracted from 25 cm^2^ tissue culture flasks using QIAzolLysis Reagent (Qiagen, Germany) according to manufacturer's instructions. The concentration and purity of the isolated RNA were determined using UV spectrophotometry (NanoDrop, Thermo Scientific, Waltham, MA, USA). The integrity of the RNA was verified using agarose gel electrophoresis stained with ethidium bromide. Reverse transcription was performed using TaqMan Reverse Transcription Reagents (Life Technologies, Carlsbad, CA, USA) using 1 *μ*g of total RNA. Real-time RT-PCR was performed using SYBR Green (Applied Biosystems, ONT, Canada, 367659). Specific sequences of the primers for endothelial NO synthase (*ENOS*), inducible NO synthase (*INOS*), catalase (*CAT*), arginase II (*ARGII*), and glyceraldehyde-3-phosphate dehydrogenase (*GAPDH*) (Exxtend Biotecnologia Ltda., Sao Paulo, Brazil) as a housekeeping gene control are shown in Table 1. The primers were designed to comprise at least one intron to minimize the noise due to genomic contamination. The PCR parameters were an initial denaturation (one cycle at 95°C for 10 min), denaturation and annealing/amplification at 95°C for 10 s and 60°C for 30 s, respectively, for 40 cycles, and a melting curve, 72°C, with the temperature gradually increasing (0.5°C) to 95°C.

### 2.8. Statistical Analysis

Continuous variables were expressed as mean and standard deviation (SD). Normality of distribution was assessed by Shapiro Wilk test. Mann-Whitney test was used to compare clinical data in 2 groups, while 2-tailed unpaired* t*-test was used to compare apoptosis and ROS content between 2 groups. Comparisons among more than 2 groups were performed using 1-way ANOVA with Tukey's post hoc test. Categorical data were presented in percentages and compared by Fisher's exact test. Spearman correlation was performed between TNF-*α* and PWV. The level of significance (*α*) accepted was less than 0.05.

## 3. Results

### 3.1. Characteristics of Study Participants

**Figure 1:**
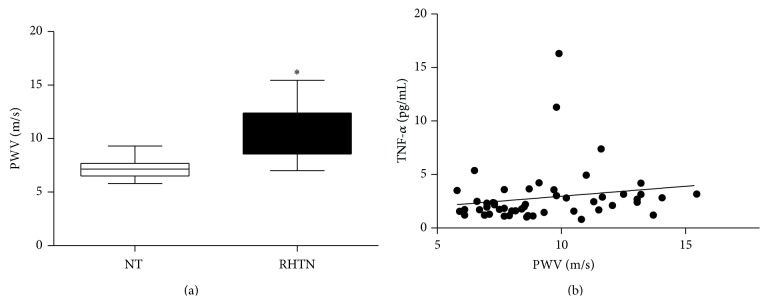
(a) Pulse wave velocity (PWV) in normotensive subjects (NT) compared to resistant hypertensive patients (RHTN) (^∗^ *p* < 0.05 versus NT). (b) Correlation between TNF-*α* levels and PWV in the whole group (*n* = 51; *r* = 0.31; *p* = 0.02).

General patients' characteristics are shown in Table 2. Normotensive and RHTN groups showed similar characteristics such as age, gender, body mass index, and systolic, diastolic, and pulse pressure. Resistant hypertensive patients showed higher levels of TNF-*α* compared to normotensive subjects (3.3 versus 2.1 pg/mL), as well as higher PWV (10.5 versus 7.2 m/s), represented in Figure 1. Statistically significant correlation was found between TNF-*α* levels and PWV measures in the whole group (Figure 1).

### 3.2. Effect of Infliximab on Endothelial Cell Apoptosis and Reactive Oxygen Species (ROS) Production

**Figure 2:**
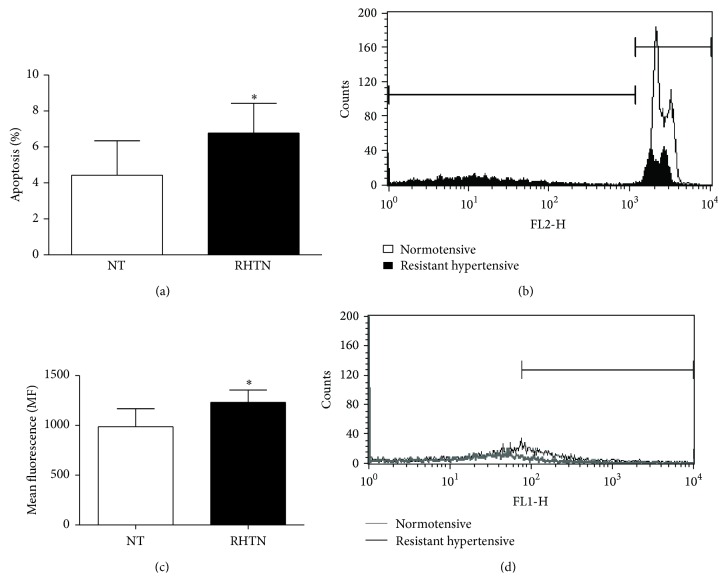
Percentage of apoptosis (a) and representative histograms overlay depicting fluorescence at FL2 (b) of HUVECs incubated with DNA fragmentation buffer and previously treated with serum from normotensive individuals (NT, *n* = 8) or resistant hypertensive patients (RHTN, *n* = 8). Mean ROS content (c) and representative histograms overlay depicting fluorescence at FL1 (d) from HUVECs incubated with DHCF and previously treated with serum from NT (*n* = 8) or RHTN (*n* = 8) (^∗^ *p* < 0.05 versus NT). Mean ± SD. The experiments were performed in duplicate.

**Figure 3:**
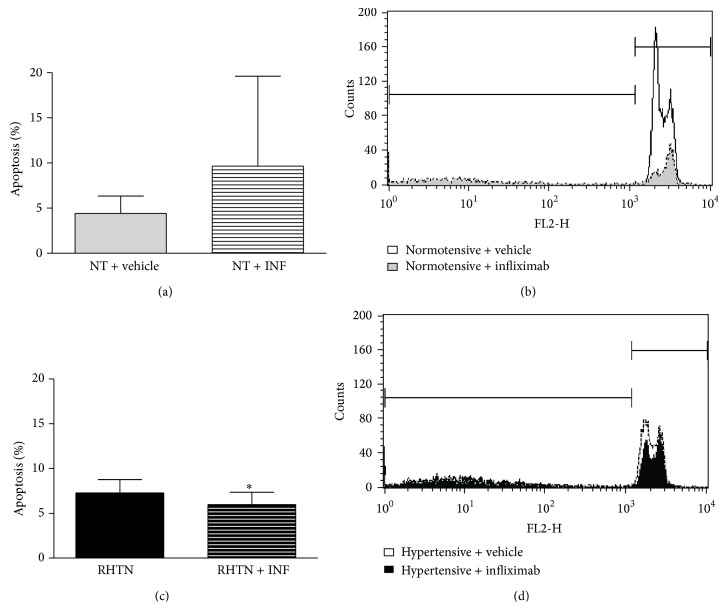
Percentage of apoptotic cells (cells with fragmented DNA) (a) and representative histograms overlay depicting fluorescence at FL2 (b) of HUVECs incubated with DNA fragmentation buffer and previously treated with serum from normotensive individuals (NT, *n* = 8) added with vehicle or infliximab. Percentage of apoptotic cells (cells with fragmented DNA) (c) and representative histograms overlay depicting fluorescence at FL2 (d) of HUVECs incubated with DNA fragmentation buffer and previously treated with serum from hypertensive patients (RHTN, *n* = 8) added with vehicle or infliximab (^∗^ *p* < 0.05 versus vehicle). Mean ± SD. The experiments were performed in duplicate.

HUVECs incubated with serum from RHTN showed increased cell apoptosis (4.43 ± 1.9 versus 7.28 ± 1.5%; *p* = 0.02) and higher reactive oxygen species (ROS) content (987 ± 181 versus 1231 ± 127 mean fluorescence values; *p* = 0.01), compared to normotensive subjects (Figure 2). The treatment with infliximab attenuated the apoptosis of HUVECs incubated with serum from RHTN (7.28 ± 1.5 versus 5.97 ± 1.39%; *p* = 0.04) but exerted no effect on ROS production. Although we observed a tendency pointing to an increase, infliximab did not change the apoptosis of HUVECs incubated with normotensive serum (4.43 ± 1.9 versus 9.66 ± 9.95; *p* = 0.13) (Figure 3).

### 3.3. Gene Expression Profile in HUVECs Incubated with Serum from Normotensive and Resistant Hypertensive Subjects

**Figure 4:**
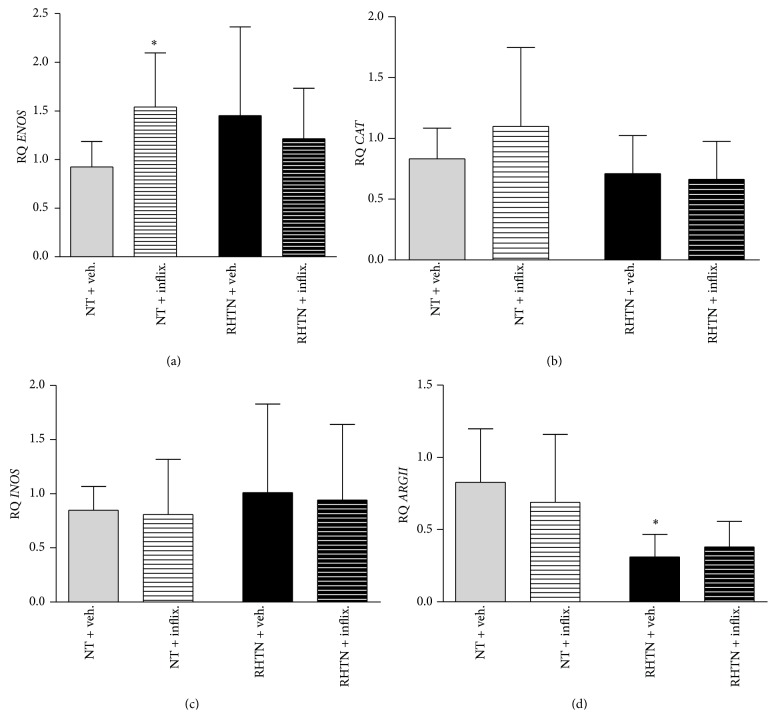
Gene expression of serum-treated HUVECs. Expression of genes normalized for GAPDH was investigated in human umbilical vein endothelial cells stimulated with plasma obtained from normotensive subjects (NT, *n* = 8) or resistant hypertensive patients (RHTN, *n* = 8) and treated with vehicle or infliximab. (a) Gene expression of endothelial nitric oxide synthase (*ENOS*). (b) Gene expression of catalase (*CAT*). (c) Gene expression of inducible nitric oxide synthase (*INOS*). (d) Gene expression of arginase II (*ARGII*) (mean ± SD, ^∗^ *p* < 0.05 versus NT vehicle). The experiments were performed in duplicate.

Normalized expression of the genes* ENOS*,* INOS*,* CAT*, and* ARGII* was shown in Figure 4. While no differences in* ENOS* expression were found between cells incubated with NT or RHTN serum, we found increased* ENOS* expression after treatment with infliximab only in the normotensive group (Figure 4(a)). No differences in* INOS* and* CAT* expression were observed among the groups. However, arginase II (*ARGII*) expression was lower in HUVECs incubated with serum of resistant hypertensive patients compared to normotensive serum, but no effect of infliximab was found in both groups.

## 4. Discussion

The present study demonstrated that resistant hypertensive subjects have higher arterial stiffness and increased TNF-*α* plasma levels compared to normotensive subjects. Moreover, TNF-*α* levels were positively correlated with carotid-femoral PWV. We also found that endothelial cells incubated with RHTN serum showed higher apoptosis rate than cells incubated with serum from normotensive subjects. The treatment with TNF-*α* inhibitor reduced apoptosis induced by RHTN serum. The inhibition of TNF-*α* increased the expression of* ENOS* in the cells incubated with normotensive serum, but no changes in* INOS*,* CAT*, and* ARGII* expression were observed. In addition, the expression of* ARGII* decreased in cells incubated with RHTN compared to NT serum.

The contributions of immune system to cardiovascular damage have been largely investigated. The lack of immune cells prevents vascular damage and the development of hypertension on several animal models of hypertension as angiotensin II infusion and DOCA-salt [3, 25]. Taken together, these findings suggest that the inhibition of inflammatory pathways may be beneficial for vascular damage prevention and treatment of hypertension. Recent study from our laboratory found that TNF-*α* inhibition reduced systolic BP and left ventricular hypertrophy and activated AKT/eNOS pathway, improving vascular function in hypertensive rats [6]. Indeed, higher levels of TNF-*α* and increased arterial stiffness were found in hypertensive subjects compared with normotensive subjects [19]. Also, arterial stiffness and TNF-*α* were positively correlated in hypertensive patients [16]. Previous studies reported that TNF-*α* plasma levels are associated with elevated blood pressure in apparently healthy subjects [1, 2].

Since RHTN patients have increased arterial stiffness and positive correlation with TNF-*α* levels, we investigated the effects of RHTN serum on endothelial cells in culture, by measuring apoptosis percentage and ROS content and whether those effects were mediated by TNF-*α*. We found that serum from RHTN promotes endothelial cells apoptosis and increases ROS content. We found reduction in cell apoptosis after the treatment with TNF-*α* inhibitor, but no changes in ROS content. Corroborating with these findings, we did not find changes in catalase gene expression after TNF-*α* inhibition. Our findings suggest that TNF-*α* may participate in apoptosis of endothelial cells but seems to not affect oxygen species.

TNF-*α* was also shown to stimulate endothelial microparticles (EMPs) releasing, which is a marker of endothelial dysfunction, and reactive oxygen species (ROS) production, which suggest that ROS are important mediators of TNF pathway [26]. Both higher ROS production and EMP releasing were associated with apoptosis. These findings are consistent with higher ROS content in HUVECs incubated with resistant hypertensive serum. Accordingly, we previously demonstrated that isoprostane levels, an oxidative stress marker, were associated with endothelial dysfunction in RHTN patients [27]. However, as we did not observe reduction in ROS content with TNF-*α* inhibition, other factors present in RHTN serum may be stimulating ROS formation.

The endothelium represents the main regulator of wall homeostasis through releasing of several molecules such as nitric oxide (NO) that is continuously produced by healthy endothelial cells. L-arginine is converted into NO by endothelial NO synthase. Also, NO can be produced in response to immunological stimuli through inducible NO synthase (iNOS). Interestingly, HUVECs incubated with RHTN serum had tendency of higher expression of eNOS compared to cells treated with NT serum, irrespective of the increased endothelial dysfunction found in those patients [9, 27]. Moreover, L-arginine is also a substrate for arginase, an enzyme expressed in the endothelium; TNF-*α* upregulates the expression of arginase in ECs, which decreases L-arginine availability to eNOS and leads to O^−2^ production. ROS production impairs vasodilatation mediated by NO [20, 28]. Surprisingly, we observed decreased arginase II (ARGII) expression in cells treated with RHTN serum compared to cells incubated with NT serum. Taken together, ECs treated with RHTN serum showed higher eNOS expression concomitantly with decreased ARGII expression. This enzymatic pattern would favor NO bioavailability and vasodilation in this* in vitro* system. Therefore, we may speculate that serum from RHTN has substances able to stimulate NO production in our* in vitro* system (functional endothelial cells) but endothelial cells of the patients may not respond to these stimuli* in vivo*, explaining the presence of endothelial dysfunction in these patients [9]. We also observed that the treatment with infliximab in cells incubated with serum from normotensive subjects displayed increased eNOS expression, suggesting that TNF-*α* might impair NO synthesis in ECs. Indeed, animal study with estrogen-deficient rats treated with anti-TNF-*α* had increased expression of tissue eNOS [29]. TNF-*α* has a crucial role in expression of eNOS suppressing eNOS mRNA and protein levels by decreasing mRNA stability [30].

On the other hand, we do not discard the possibility that TNF-*α* may modulate other NO synthases, as inducible NO synthase (iNOS), promoting nitrosative stress and endothelial dysfunction [31]; however, we found no statistical differences in iNOS expression between the groups or after infliximab treatment. We observed a large variability in HUVECs gene expression incubated with serum from different hypertensive subjects, which may reflect the differences between patients including severity and time of hypertension, presence of comorbidities, and differences in drug regimens.

The treatment of inflammatory disease (anti-neutrophil cytoplasmic antibody-associated systemic vasculitis) with infliximab improved endothelial function [32]; thus, TNF-*α* inhibitors have an emerging role in prevention of vascular dysfunction. Furthermore, arterial stiffness was improved in patients with rheumatoid arthritis treated with TNF-*α* inhibitors [21]. Further studies are needed to elucidate the mechanisms of the proinflammatory cytokine TNF-*α* on arterial stiffness. In conclusion, serum TNF-*α* in resistant hypertensive subjects induces apoptosis in human endothelial cells. These results suggest that TNF-*α* might mediate, at least in part, vascular injury in resistant hypertension. Finally, future clinical trials with TNF-*α* inhibitors in these patients may represent a field of interest.

## Figures and Tables

**Table 1:** Primers sequences.

Name	Forward primer (sense) 5′ → 3′	Reverse primer (antisense) 5′ → 3′
Human* GAPDH *	GTTAGGAAAGCCTGCCGGT	AGTTAAAAGCAGCCCTGGTGA
Human *ENOS *	GTGGCTGTCTGCATGGACCT	CCACGATGGTGACTTTGGCT
Human *INOS *	GATCAAAAACTGGGGCAGCG	CTCATCTGGAGGGGTAGGCT
Human *ARGII *	TTAGCAGAGCTGTGTCAGATGGCT	GGGCATCAACCCAGACAACACAAA
Human *CAT *	ACAGCAAACCGCACGCTA	CACGGGGCCCTACTGTAATAA

*GAPDH*: glyceraldehyde-3-phosphate dehydrogenase, *ENOS*: endothelial nitric oxide (NO) synthase, *INOS*: inducible NO synthase, *ARGII*: arginase II, and* CAT*: catalase.

**Table 2:** General characteristics of normotensive and resistant hypertensive subjects.

	NT (*n* = 19)	RHTN (*n* = 32)	*p *value
Age (years)	52 ± 5.0	57 ± 13	0.06
Gender (F/M)	9/10	19/13	0.56
BMI (kg/m^2^)	25.1 ± 2.3	26.3 ± 2.9	0.14
Office SBP (mmHg)	121 ± 14	146 ± 16^∗^	<0.0001
Office DBP (mmHg)	78 ± 8	86 ± 14^∗^	0.04
Office PP (mmHg)	43 ± 9	61 ± 13^∗^	<0.0001
PWV (m/s)	7.2 ± 1.0	10.5 ± 2.2^∗^	<0.001
TNF-*α* (pg/mL)	2.1 ± 1.2	3.3 ± 3.1^∗^	0.04

Mean ± SD. NT: normotensive subjects; RHTN: resistant hypertensive patients; BMI: body mass index; SBP: systolic blood pressure; DBP: diastolic blood pressure; PP: pulse pressure; PWV: pulse wave velocity; TNF-*α*: tumoral necrosis factor-*α*. ^∗^ *p* < 0.05 versus NT.

## References

[1] Bautista L. E., Vera L. M., Arenas I. A., Gamarra G. (2005). Independent association between inflammatory markers (C-reactive protein, interleukin-6, and TNF-*α*) and essential hypertension. *Journal of Human Hypertension*.

[2] Ito H., Ohshima A., Tsuzuki M. (2001). Association of serum tumour necrosis factor-*α* with serum low-density lipoprotein-cholesterol and blood pressure in apparently healthy Japanese women. *Clinical and Experimental Pharmacology and Physiology*.

[3] Guzik T. J., Hoch N. E., Brown K. A. (2007). Role of the T cell in the genesis of angiotensin II-induced hypertension and vascular dysfunction. *Journal of Experimental Medicine*.

[4] Lee D. L., Sturgis L. C., Labazi H. (2006). Angiotensin II hypertension is attenuated in interleukin-6 knockout mice. *The American Journal of Physiology—Heart and Circulatory Physiology*.

[5] Marvar P. J., Thabet S. R., Guzik T. J. (2010). Central and peripheral mechanisms of T-lymphocyte activation and vascular inflammation produced by angiotensin II-induced hypertension. *Circulation Research*.

[6] Filho A. G., Kinote A., Pereira D. J. (2013). Infliximab prevents increased systolic blood pressure and upregulates the AKT/eNOS pathway in the aorta of spontaneously hypertensive rats. *European Journal of Pharmacology*.

[7] Yoshida S., Takeuchi T., Kotani T. (2014). Infliximab, a TNF-*α* inhibitor, reduces 24-h ambulatory blood pressure in rheumatoid arthritis patients. *Journal of Human Hypertension*.

[8] Armario P., Oliveras A., Hernández Del Rey R., Ruilope L. M., De La Sierra A. (2011). Prevalence of target organ damage and metabolic abnormalities in resistant hypertension. *Medicina Clinica*.

[9] Figueiredo V. N., Yugar-Toledo J. C., Martins L. C. (2012). Vascular stiffness and endothelial dysfunction: correlations at different levels of blood pressure. *Blood Pressure*.

[10] Laurent S., Boutouyrie P., Asmar R. (2001). Aortic stiffness is an independent predictor of all-cause and cardiovascular mortality in hypertensive patients. *Hypertension*.

[11] Harrison D. G., Gongora M. C. (2009). Oxidative stress and hypertension. *Medical Clinics of North America*.

[12] Rajagopalan S., Kurz S., Münzel T. (1996). Angiotensin II-mediated hypertension in the rat increases vascular superoxide production via membrane NADH/NADPH oxidase activation: contribution to alterations of vasomotor tone. *Journal of Clinical Investigation*.

[13] Patel R., Cardneau J. D., Colles S. M., Graham L. M. (2006). Synthetic smooth muscle cell phenotype is associated with increased nicotinamide adenine dinucleotide phosphate oxidase activity: effect on collagen secretion. *Journal of Vascular Surgery*.

[14] Madhur M. S., Funt S. A., Li L. (2011). Role of interleukin 17 in inflammation, atherosclerosis, and vascular function in apolipoprotein e-deficient mice. *Arteriosclerosis, Thrombosis, and Vascular Biology*.

[15] Park S., Lakatta E. G. (2012). Role of inflammation in the pathogenesis of arterial stiffness. *Yonsei Medical Journal*.

[16] Mahmud A., Feely J. (2005). Arterial stiffness is related to systemic inflammation in essential hypertension. *Hypertension*.

[17] Wu J., Thabet S. R., Kirabo A. (2014). Inflammation and mechanical stretch promote aortic stiffening in hypertension through activation of p38 mitogen-activated protein kinase. *Circulation Research*.

[18] Madhur M. S., Lob H. E., McCann L. A. (2010). Interleukin 17 promotes angiotensin II-induced hypertension and vascular dysfunction. *Hypertension*.

[19] Barbaro N. R., Fontana V., Modolo R. (2015). Increased arterial stiffness in resistant hypertension is associated with inflammatory biomarkers. *Blood Press*.

[20] Murdaca G., Spanò F., Cagnati P., Puppo F. (2013). Free radicals and endothelial dysfunction: potential positive effects of TNF-*α* inhibitors. *Redox Report*.

[21] Dulai R., Perry M., Twycross-Lewis R., Morrissey D., Atzeni F., Greenwald S. (2012). The effect of tumor necrosis factor-*α* antagonists on arterial stiffness in rheumatoid arthritis: a literature review. *Seminars in Arthritis & Rheumatism*.

[22] Moreau K. L., Deane K. D., Meditz A. L., Kohrt W. M. (2013). Tumor necrosis factor-*α* inhibition improves endothelial function and decreases arterial stiffness in estrogen-deficient postmenopausal women. *Atherosclerosis*.

[23] Mäki-Petäjä K. M., Elkhawad M., Cheriyan J. (2012). Anti-tumor necrosis factor-*α* therapy reduces aortic inflammation and stiffness in patients with rheumatoid arthritis. *Circulation*.

[24] Calhoun D. A., Jones D., Textor S. (2008). Resistant hypertension: diagnosis, evaluation, and treatment. A scientific statement from the American Heart Association Professional Education Committee of the Council for High Blood Pressure Research. *Hypertension*.

[25] Wenzel P., Knorr M., Kossmann S. (2011). Lysozyme M-positive monocytes mediate angiotensin ii-induced arterial hypertension and vascular dysfunction. *Circulation*.

[26] Lee S. K., Yang S., Kwon I., Lee O., Heo J. H. (2014). Role of tumour necrosis factor receptor-1 and nuclear factor-*κ*B in production of TNF-*α*-induced pro-inflammatory microparticles in endothelial cells. *Thrombosis and Haemostasis*.

[27] de Faria A. P. C., Fontana V., Modolo R. (2014). Plasma 8-isoprostane levels are associated with endothelial dysfunction in resistant hypertension. *Clinica Chimica Acta*.

[28] Gao X., Xu X., Belmadani S. (2007). TNF-*α* contributes to endothelial dysfunction by upregulating arginase in ischemia/reperfusion injury. *Arteriosclerosis, Thrombosis, and Vascular Biology*.

[29] Arenas I. A., Armstrong S. J., Xu Y., Davidge S. T. (2005). Chronic tumor necrosis factor-*α* inhibition enhances NO modulation of vascular function in estrogen-deficient rats. *Hypertension*.

[30] Lee K. S., Kim J., Kwak S. N. (2014). Functional role of NF-*κ*B in expression of human endothelial nitric oxide synthase. *Biochemical and Biophysical Research Communications*.

[31] Oliveira-Paula G. H., Lacchini R., Tanus-Santos J. E. (2014). Inducible nitric oxide synthase as a possible target in hypertension. *Current Drug Targets*.

[32] Booth A. D., Jayne D. R. W., Kharbanda R. K. (2004). Infliximab improves endothelial dysfunction in systemic vasculitis: a model of vascular inflammation. *Circulation*.

